# Cellular and molecular mechanisms of mitochondrial function

**DOI:** 10.1016/j.beem.2012.05.003

**Published:** 2012-12

**Authors:** Laura D. Osellame, Thomas S. Blacker, Michael R. Duchen

**Affiliations:** a Department of Cell and Developmental Biology, University College London, London WC1E 6BT, United Kingdom; b UK Parkinson’s Disease Consortium, Institute of Neurology, University College London, London WC1N 3BG, United Kingdom; c Department of Physics and Astronomy, University College London, London WC1E 6BT, United Kingdom

**Keywords:** mitochondria, oxidative phosphorylation, apoptosis, intracellular calcium, mitochondrial fission

## Abstract

Mitochondria are membrane bound organelles present in almost all eukaryotic cells. Responsible for orchestrating cellular energy production, they are central to the maintenance of life and the gatekeepers of cell death. Thought to have originated from symbiotic ancestors, they carry a residual genome as mtDNA encoding 13 proteins essential for respiratory chain function. Mitochondria comprise an inner and outer membrane that separate and maintain the aqueous regions, the intermembrane space and the matrix. Mitochondria contribute to many processes central to cellular function and dysfunction including calcium signalling, cell growth and differentiation, cell cycle control and cell death. Mitochondrial shape and positioning in cells is crucial and is tightly regulated by processes of fission and fusion, biogenesis and autophagy, ensuring a relatively constant mitochondrial population. Mitochondrial dysfunction is implicated in metabolic and age related disorders, neurodegenerative diseases and ischemic injury in heart and brain.

## Introduction

Mitochondria are integral to normal cellular function as they are responsible for energy production in eukaryotes, including the synthesis of phospholipids and heme, calcium homeostasis, apoptotic activation and cell death.^1,2^ Alterations in mitochondrial function often associate with disease states including endocrine related disorders such as diabetes mellitus, reflecting the centrality of energy homeostasis in beta cell physiology.^3^ Mitochondria have retained their own genome, reflecting a bacterial evolutionary origin and while the majority of mitochondrial proteins are encoded by nuclear genes, a few respiratory proteins and mitochondrial tRNA’s remain encoded by the mitochondrial genome.^4^ Mitochondrial biogenesis requires coordination of nuclear and mitochondrial encoded gene expression in order to ensure the correct assembly and function of a large set of proteins that comprise the mitochondrial respiratory chain.^5^ Defects in mitochondrial DNA (mtDNA) result in an array of diseases which include Leigh syndrome, Leber’s hereditary optic neuropathy, MELAS (Mitochondrial Encephalomyopathy with Lactic Acidosis and Stroke like episodes) and MERFF (Myoclonus Epilepsy with Ragged Red Fibres).^6,7^ Diseases that result from mutations of mtDNA do not follow Mendelian inheritance but are instead inherited through the maternal line, and show variable severity of expression reflecting the heteroplasmy of the mtDNA population – in which a mixture of wild type and mutant mtDNA coexist. In most of these diseases a higher mutant load is generally associated with more severe manifestations of disease. These disorders tend to give rise to pathology in tissues that are reliant on absolute mitochondrial function, in particular oxidative phosphorylation and with little capacity to upregulate compensatory increases in glycolysis, suggesting a direct correlation between efficient energy production and mitochondrial function.^8^

Oxygen is used to support mitochondrial respiration which in turn is used to build a proton gradient across the expanded surface area of the cristae. It is the proton gradient, expressed largely as a mitochondrial transmembrane potential gradient, that then drives much of mitochondrial physiology – the synthesis of adenosine triphosphate (ATP), the transfer of calcium and other ion exchangers, and the import of proteins.^9^ Oxidative phosphorylation is then regulated by cross-talk with cellular calcium signalling, so that the transfer of Ca^2+^ into the mitochondrial matrix signals an increased energy demand and drives an increased energy provision by oxidative phosphorylation by upregulating the rate limiting enzymes of the citric acid cycle.^10^ Bioenergetic failure is often fatal to cellular function, in particular in muscle and nerve cells. Excessive mitochondrial Ca^2+^ uptake is of particular importance in the brain, heart and muscle, where prolonged unphysiological increases in Ca^2+^ influx, especially when combined with oxidative stress, may result in a pathological transformation – the opening of the mitochondrial permeability transition pore (mPTP) and induction of necrotic cell death.^1^ Indeed, mitochondria have been referred to as ‘poison cabinets’ of the cell as key proteins released from the matrix and inner membrane initiate apoptotic cell death. Thus, mitochondria are positioned at the heart of cell life and cell death so that cellular function often mirrors the degree of mitochondrial viability, and impaired mitochondrial function is increasingly associated with disease.

## Mitochondrial bioenergetics

**Fig. 1:**
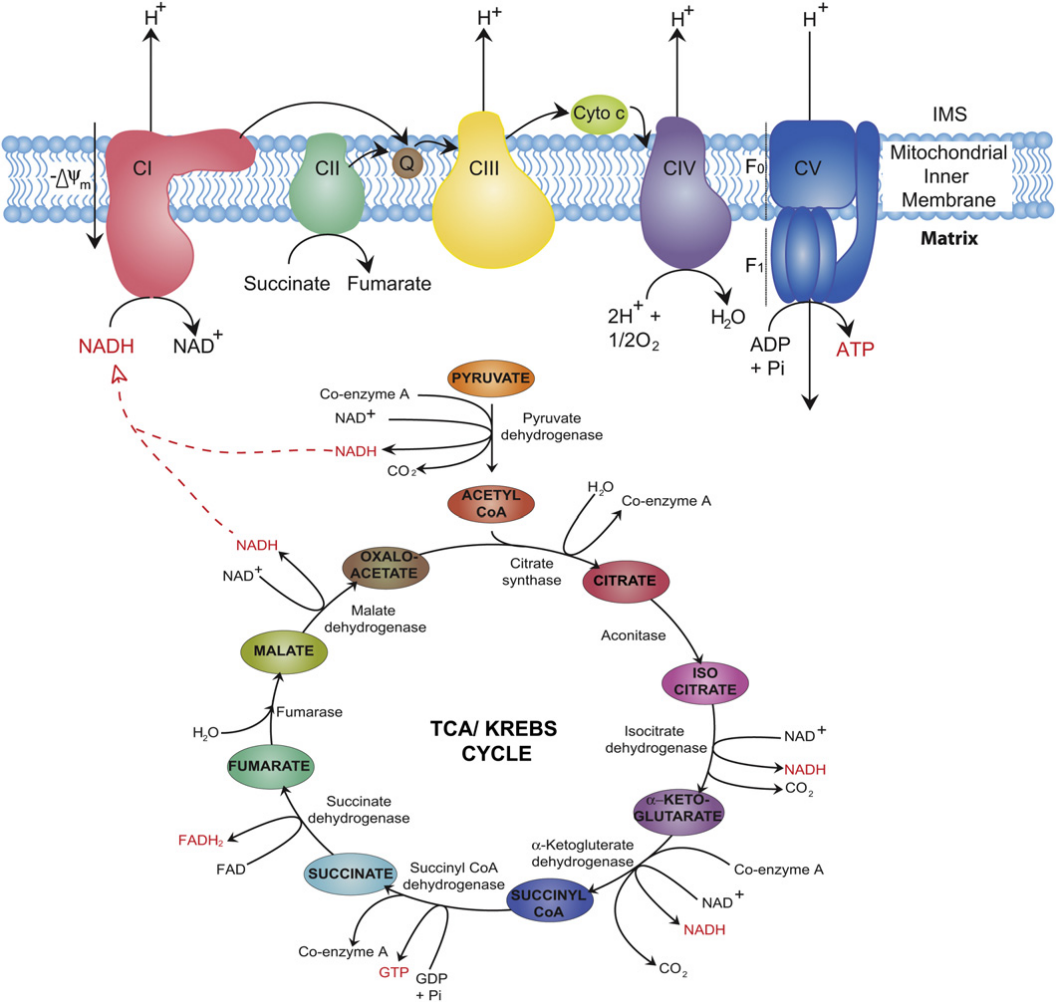
Bioenergetics of the electron transport chain and the TCA/Kerbs cycle. Pyruvate is converted to high-energy molecules LIKE NADH, GTP and FADH_2_ through catalyzation by TCA/Kerbs cycle enzymes. NADH generated is shuttled to complex I and is converted to NAD^+^ driving oxidative phosphorylation. Transfer of electrons through the chain maintains the membrane potential via proton pumping into the IMS. In this final step ADP is phosphorylated to form ATP via complex V (ATP synthase).

The mitochondria house the major enzymatic systems used to complete the oxidation of sugars, fats and proteins to produce usable energy in the form of ATP^9^ (Fig. 1). Each of these three substrates can be catabolised to acetyl-CoA, which then enters the first of these processes: the citric acid cycle, taking place in the mitochondrial matrix. Sugars enter the mitochondria as pyruvate after undergoing glycolysis in the cytosol. Pyruvate dehydrogenase facilitates its conversion to acetyl-CoA. Beta oxidation converts fatty acids to acetyl-CoA inside the mitochondria, while various enzymes exist for the conversion of specific amino acids into pyruvate, acetyl-CoA or directly into particular citric acid cycle intermediates.^11,12^

In the citric acid cycle, also known as the tricarboxylic acid (TCA) or Krebs cycle, the two-carbon acetyl group of acetyl-CoA is transferred to the four-carbon oxaloacetate, forming the six-carbon molecule citrate. In a series of seven subsequent enzymatic steps, the citrate is oxidized back to oxaloacetate, with the excess carbon carried away as two molecules of carbon dioxide and the electrons removed in the process passed to the cofactors nicotinamide adenine dinucleotide(NADH) and flavin adenine dinucleotide (FADH_2_). The oxaloacetate is now free to participate in the cycle again, while the free energy liberated is carried by NADH and FADH_2_ to the mitochondrial electron transport chain.

Also known as the respiratory chain, the electron transport chain consists of a series of multisubunit protein complexes embedded in the inner mitochondrial membrane (Fig. 1). Here, the electrons removed from the citric acid cycle by NADH and FADH_2_ are used to power the pumping of protons from the matrix to the intermembrane space, generating a potential difference across the inner mitochondrial membrane. This potential difference is ultimately used to power the synthesis of ATP in the final step of oxidative phosphorylation.

NADH brings free energy to the electron transport chain by binding to the largest of the respiratory complexes, NADH dehydrogenase, or complex I. This L-shaped enzyme contains a hydrophobic domain embedded in the inner mitochondrial membrane and a hydrophilic arm, protruding into the mitochondrial matrix, containing the NADH binding site. The whole complex consists of 45 subunits and is almost 1 MDa in mass. The expression of specific subunits varies between tissues and, at present, the functional significance of most of these subunits is relatively unknown.^13^ NADH donates two electrons, carried away from the citric acid cycle, to a flavin mononucleotide prosthetic group contained in the hydrophilic arm of complex I. These electrons are then passed down the arm via a series of iron–sulphur clusters to the lipid soluble redox carrier coenzyme Q.

Linked to the passage of electrons from NADH through the complex is the translocation of four protons from the matrix across the inner mitochondrial membrane. Recent studies of complex I from *Thermus thermophilus* and *Escherichia coli* have highlighted a mechanism for proton translocation whereby the electron transfer induces a conformational change in the hydrophilic arm. These mechanical stresses are then passed to the hydrophobic domain, causing a reconfiguration of the protein and associated pumping of protons into the intermembrane space.^14^

While NADH must diffuse to complex I in order to feed the electrons it ferries into the electron transport chain, the enzyme catalysing the reduction of FAD to FADH_2_ in the citric acid cycle, succinate dehydrogenase, is itself part of the electron transport chain. Also known as complex II, this 123 kDa enzyme, like complex I, is located on the inner mitochondrial membrane and contains FAD as a prosthetic group alongside iron–sulphur clusters to aid the passing of the donated electrons to coenzyme Q.^15^ No protons are pumped from the mitochondrial matrix by this complex, which is unique amongst the respiratory chain complexes as being entirely encoded by nuclear DNA.

Coenzyme Q, reduced by either complex I or complex II, is able to freely diffuse through the inner mitochondrial membrane to donate its electrons to the third complex of the electron transport chain, cytochrome *c* reductase. This enzyme, the smallest of the four electron transport complexes, oxidizes coenzyme Q and passes the liberated electrons to two molecules of cytochrome *c*, a 13 kDa water-soluble redox protein that also plays a key role in apoptosis.^16^ Two protons obtained from the oxidation of coenzyme Q are deposited in the intermembrane space, and an additional two protons are translocated from the mitochondrial matrix.^17^

The ultimate fate of the electrons passed along the chain is in the conversion of oxygen to water. This occurs at complex IV, cytochrome *c* oxidase. Four molecules of cytochrome *c* donate one electron each to the enzyme’s iron/copper active site, where the production of two H_2_O molecules from one O_2_ molecule is then catalysed. Again, alongside this reaction, four protons are pumped from the mitochondrial matrix into the intermembrane space.^18^

As the electrons travel through the electron transport chain, their free energy decreases alongside the steady increase in redox potential of their carriers, finally ending with oxygen with the largest redox potential of all. The energy released during the electron’s traversal down the free energy “staircase” is the power source for the thermodynamically unfavourable pumping of protons against their concentration gradient occurring at complexes I, III and IV.

Following the citric acid cycle and the electron transport chain, all that remains for the conversion of the energy stored in the chemical bonds of substrates into the ubiquitous “energy currency” ATP is the coupling of this approximately 200 mV membrane voltage to the phosphorylation of adenosine diphosphate (ADP).^19^ This coupling was proposed by Peter Mitchell in 1961, for which he was awarded the Nobel Prize in Chemistry in 1978.^20^ The enzyme responsible for the final step of mitochondrial oxidative phosphorylation is ATP synthase (complex V). It consists of two domains – the F_0_ domain spans the inner mitochondrial membrane while the F_1_ domain drops into the mitochondrial matrix – giving the enzyme its alternate name of F_0_F_1_ ATPase.

The mechanism by which ATP synthase functions was first demonstrated by Paul Boyer and John Walker, resulting in their award of the 1997 Nobel Prize in Chemistry “for their elucidation of the enzymatic mechanism underlying the synthesis of ATP”. In this scheme, ATP synthase acts as a rotary molecular motor. An elongated peripheral stalk anchors the head of the F_1_ domain to the inner mitochondrial membrane to form the stator. The transmembrane proton channel of the F_0_ domain and an asymmetric stalk protruding inside the head of the F_1_ domain form the rotor. The static head of the F_1_ domain has a quasi-3-fold rotational symmetry, with each element containing a binding site for ADP and phosphate. As protons deposited in the intermembrane space by the electron transport chain flow down their electrochemical gradient through the F_0_ domain, the rotor turns inside the head of the stator. As the rotating stalk passes each binding site in turn, conformational changes are induced that make the combination of bound ADP and phosphate into ATP energetically favourable.^21^ Thus, each turn of the rotor produces 3 molecules of ATP.

Estimates of the number of protons required to pass through ATP synthase in order to produce a single ATP molecule vary between 2 and 5.^22^ Extrapolating this in order to gauge how many NADH or FADH_2_ molecules are required to produce a single ATP is difficult because protons are able to leak across the mitochondrial membranes, dissipating their energy as heat. The removal of protons from the intermembrane space through avenues other than ATP synthase is known as uncoupling. Estimates of 3 ATP molecules produced by oxidative phosphorylation per molecule of NADH and 2 ATP molecules per molecule of FADH_2_ are typical. Fewer ATP molecules result from the oxidation of FADH_2_ as complex II does not translocate any protons.

In addition to proton leakage uncoupling the membrane voltage from ATP production, electrons are able to leak from the electron transport chain complexes. Premature leakage of electrons allows their passage directly to oxygen, rather than being passed to oxygen to form water at complex IV, producing superoxide. Superoxide is highly reactive and, as such, highly toxic to the cell, imparting so-called “oxidative stress”. Oxidative stress has been implicated in many pathologies ranging from atherosclerosis and diabetes to neurodegenerative disease and cancer, and is thought to play a major role in ageing.^23^

## Mitochondrial calcium signalling

Mitochondria are in constant communication with the cytosol to coordinate the balance between the energy demands of the cell and energy production by oxidative phosphorylation. This is primarily orchestrated through calcium signalling between the cytosol and matrix. Cellular Ca^2+^ signalling is fundamental to most forms of cellular ‘activation states’: Ca^2+^signals govern most processes that are associated with increased demands for energy – secretion, contraction, motility, electrical excitability – all of which require increased energy provision and are usually associated with a rise in cytosolic Ca^2+^ concentration ([Ca^2+^]_c_). Mitochondria express a Ca^2+^ uptake pathway, the mitochondrial calcium uniporter (MCU), which is a Ca^2+^ selective channel in the inner mitochondrial membrane.^24–26^ A rise in local [Ca^2+^]_c_ will promote mitochondrial Ca^2+^ uptake as Ca^2+^ moves down its electrochemical potential gradient into the matrix. The rise in matrix Ca^2+^ concentration ([Ca^2+^]_m_) activates the three rate limiting enzymes of the TCA cycle – pyruvate, α-ketogluterate (also called oxogluterate)and NAD-isocitrate dehydrogenases. The ATP synthase also appears to be upregulated by a rise in [Ca^2+^]_m_ although the mechanism remains unclear. These processes work together to drive an increase in NADH provision to the respiratory chain, an increase in respiration and, ultimately, an increase in the rate of ATP synthesis.^27,28^ The rise in [Ca^2+^]_c_ also activates the glutamate/aspartate transporter (ARALAR) on the inner mitochondrial membrane increasing substrate supply, a pathway that does not require specific mitochondrial Ca^2+^uptake and so is independent of mitochondrial bioenergetic competence.^29^Thus these pathways work together to match energy supply and demand in an elegant and simple manner. In most cells, the efflux of Ca^2+^from the mitochondrial matrix, driven by a Na^+^/Ca^2+^exchanger is relatively slow so that the change in [Ca^2+^]_m_ far outlasts the change in [Ca^2+^]_c_, and the metabolic response probably matches the time course of the change in [Ca^2+^]_m_.^30^ The matching of energy supply and demand happens over a longer timescale at least in muscle, where exercise is associated with a Ca^2+^mediated increase in mitochondrial biogenesis.^31^ Again, activity, and so increased energy demand, is signalled by [Ca^2+^]_c_ signals which interact through CAMKKβ to activate PGC1α and so drive an increase in mitochondrial mass in muscle. There is no data as far as we know about how these pathways operate in other tissues.

## Mitochondrial morphology

Mitochondrial bioenergetics seems strongly dependent on mitochondrial morphology – changes in morphology seem to impact on bioenergetic state, whilst changes in bioenergetics often result in changes in morphology. Mitochondrial shape is largely determined by a balance between fission and fusion events and this equilibrium maintains steady state mitochondrial morphology, mtDNA (nucleoid) and metabolic mixing, bioenergetic functionality and organelle number.^32^ The importance of fission and fusion homeostasis has been highlighted by a number of disease states linked to mutations involving shaping proteins, so that an imbalance in fission and fusion events leads to a distinct shift in the morphology and viability of the organelle.^33^

### Mitochondrial fission

Mitochondrial division is essential for organelle biogenesis and inheritance and left unregulated can lead to a heterogeneous population of organelles with non-uniform mtDNA distribution, varied ability to produce ATP, increased capacity to generate reactive oxygen species and increased susceptibility of cells to undergo apoptosis.^34^ Fission is also required for the removal of aged or damaged mitochondria through a specialized form of autophagy, termed mitophagy.^35^ This ensures that defective mitochondria are small enough to be encapsulated by lytic vesicles, autophagosomes, so that organelle content can be degraded or recycled. Defects in mitophagy are linked with autosomal recessive Parkinson’s disease though mutations in PINK1, a mitochondrial kinase, and the cytosolic E3 ubquitin ligase Parkin.^36,37^ These mutations are associated with broad mitochondrial dysfunction including alterations in mitochondrial morphology. While a number of different proteins have recently been proposed to actively contribute to the fission process, only two proteins are conserved though evolution, Dynamin related protein 1 (Drp1) and Fis1.^38–40^

**Fig. 2:**
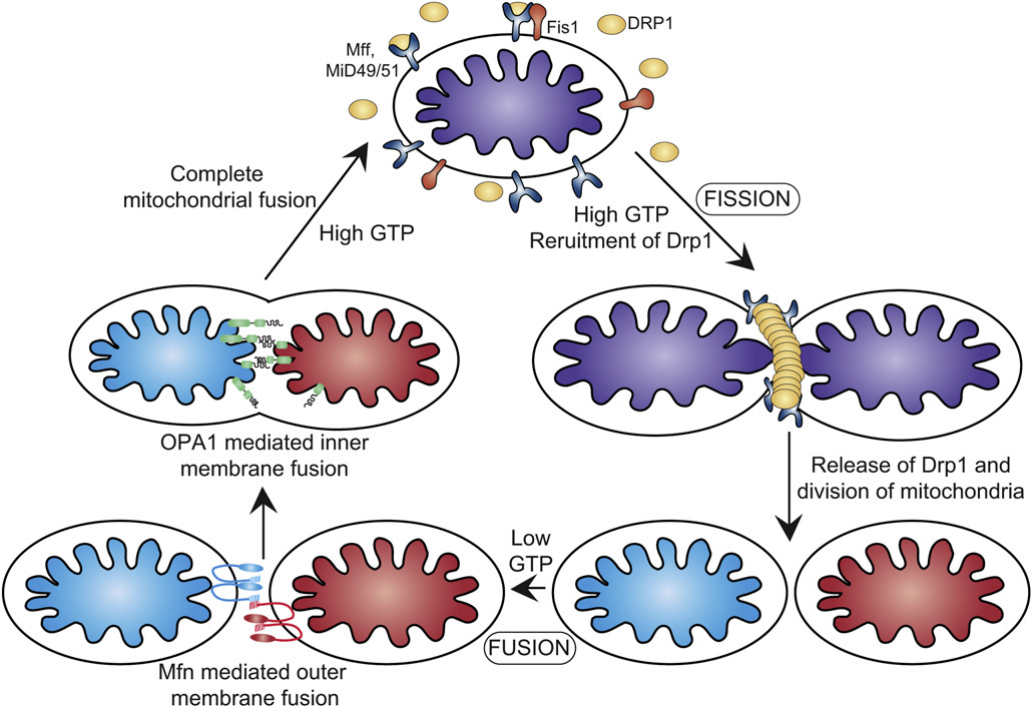
Mitochondrial dynamics. Steady state mitochondrial morphology requires a balance of fission and fusion events. Organelle division is mediated by Drp1 which forms high molecular weight oligomers on the mitochondrial surface. Once Drp1 is released fission is complete. Mitochondrial fusion is a two-step process that requires outer and inner membrane fusion. Outer membrane fusion is facilitated by mitofusin tethering of adjacent membranes. In high GTP environments, OPA1 isoforms allow inner membrane fusion.

Recent studies have highlighted the absolute reliance on Drp1 activity and have illustrated how aberrant mitochondrial morphology affects viability at the level of the whole organism. A patient harbouring a dominant-negative Drp1 allele presented with broad metabolic defects, abnormal brain development, optic atrophy and died 37 days after birth.^41^ In addition Drp1 knockout in mice results in developmental abnormalities in the forebrain and is ultimately embryonic lethal.^42^ Drp1 belongs to the large family of dynamin related GTPases, is dispersed in the cytosol and cycles on and off mitochondria in a GTP dependent manner (Fig. 2). It assembles on the outer membrane in multimeric ring-like structures to facilitate scission of the double membrane.^43,44^ New evidence has emerged regarding the outer membrane proteins that regulate Drp1 action at the mitochondrial surface and in turn control mitochondrial functionality.^39,40^ Much doubt has been cast on the proposed role of Fis1 as a bonafide Drp1 receptor in mammalian systems. It is widely accepted that Fis1 is the receptor in yeast, however, this mechanism does not appear to have been conserved throughout evolution, nor do adaptor proteins Cav4p and Mdv1p.^45^ Knockdown of Fis1 in mammalian systems does not inhibit the recruitment of Drp1 to the surface of the mitochondria,^46^ suggesting that there are other factors governing sequestration of Drp1. Indeed a number of new outer membrane proteins (Mitochondrial fission factor and Mitochondrial dynamics proteins 49 and 51) have recently been reported to regulate Drp1 recruitment in higher eukaryotes.^39,40^ Although these findings have added to the knowledge and complexity of the fission complex, there is still much to learn about intricate details of double membrane division. Addressed recently was the paradox regarding the size of Drp1 oligomers versus the breadth of the mitochondrial thread. In vitro assembly of Drp1 results in formation of helices that are smaller than the diameter of mitochondria, suggesting that additional factors are required to constrict the organelle.^47^ The role of the endoplasmic reticulum (ER) in facilitation of mitochondrial fission has recently been described, where the ER is responsible for the initial construction of the mitochondria.^48^ The ER:mitochondrial contacts mark the site for future Drp1 assembly and subsequent completion of the division process.^48^

### Mitochondrial fusion

Mitochondrial fusion is essential to maintain a homogenous organelle population and ensures inter-complementation of mtDNA. Mitochondrial fusion is a two-step process, in which the outer and inner membranes fuse by separate events. In mammals outer membrane fusion is controlled by two large membrane GTPase proteins, Mitofusin1 (Mfn1) and Mitofusin2 (Mfn2), whereas inner membrane fusion is controlled by Optic atrophy 1 (OPA1). It is unknown how the fusion machineries are activated and how mitochondrial content, distribution and division timing are coordinated.

The Mitofusins are essential for mitochondrial fusion; loss of mitofusin function results in fragmentation of the mitochondrial network.^49^ Of note, mutations in Mfn2 cause the autosomal dominant peripheral neuropathy, Charcot Marie-Tooth disease 2A, while reduced expression of the protein has been found in diabetic and obese patients.^50,51^ Mice lacking either Mfn1 or Mfn2 die midgestation while embryonic stem cell lines derived from mice lack any detectable fusion activity highlighting the severity of the deletions.^49^ The Mitofusins are outer membrane proteins that span the bilayer twice, leaving both the C-terminal coiled-coil domain and the N-terminal GTPase domain exposed to the cytosol (Fig. 2).^52^ The fusion mechanism is mediated by the heptad repeat (HR2) regions located in the carboxyl end of the proteins, these form oligomers by assembling a dimeric antiparallel coiled-coil.^53^ These heterotypic and homotypic dimers assemble in trans, between adjacent mitochondria and function as a mitochondrial tethering complex prior to organelle fusion.^53^Consistent with this, Mfn2 has also been implicated in tethering mitochondria to the ER. Mfn2 is enriched at contact sites between mitochondria and ER and this association is mediated by Mfn2 homotypic and heterotypic interactions.^54^ This loss of Mfn2 results in increased distance between the two organelles and impaired mitochondrial calcium uptake.^54^ This mechanistic link provides insight into the spatio-temporal relationship between the ER and mitochondria, and calcium efflux from the ER and subsequent uptake into the mitochondria, suggesting a divergent role for Mfn2 in organelle morphology.

OPA1 is a dynamin-like protein that resides in the mitochondrial inner membrane and is responsible for the maintenance of cristae morphology and inner membrane fusion with mutations resulting in optic atrophy.^55^ OPA1 consists of 8 isoforms and the steady state morphology of the organelle is dependent on the balance of long (L) and short (S) isoforms.^56^ OPA1 is cleaved by a number of processing peptidases/proteases located within the mitochondria, namely presenilin associated rhomboid-like protease, AFG3L1/2, paraplegin, OMA1 and Yme1.^56–58^ The ability of OPA1 to self-interact and oligomerize requires both L-OPA1 and S-OPA isoforms. Regulation of OPA1 cleavage by different proteases is highly dependent on access to processing sites and cellular stress stimuli. Increased levels of S-OPA1 (following processing) occur due to loss of membrane potential/induction of apoptosis; as such the mitochondrial network becomes extensively fragmented.

## Mitochondria and cell death

In many models of cell injury or disease, the irreversibility of cell injury is primarily determined by aspects of mitochondrial biology. Cell death is broadly classified as apoptotic or necrotic – programmed or accidental – although the boundaries between forms of cell death are not always so clearly defined. Apoptotic cell death plays a crucial role in early development and later in life, in removing cells that are damaged without the energy loss associated with necrotic cell death. Apoptosis is an energy dependent, active and coordinated process while necrosis is typically the result of a metabolic failure leading to energetic collapse, breakdown of ion gradients, cell swelling and structural disorganization.

A major mechanism driving necrotic cell death is opening of the mPTP. Pore opening is implicated in an ever increasing array of disease states in many different tissues, although the strongest experimental case probably lies in cell death during ischaemia and reperfusion injury in the heart. This is important and exciting as the pore is a viable therapeutic target and so identification of its involvement carries with it implications of therapeutic opportunities.

First described by Hunter and Hapworth, the abrupt loss of the mitochondrial permeability barrier following additions of Ca^2+^ or pro oxidants was later shown to result from the opening of a large conductance pore in the inner mitochondrial membrane large enough to entrap deoxyglucose.^59^ Pore opening causes collapse of the mitochondrial membrane potential, ATP depletion and the rapid progression to cell death. It has been suggested that the pore is generated by a transformation of membrane proteins with other ‘normal’ functions into a pore forming configuration – a favoured candidate has been the adenine nucleotide translocase (ANT), as this protein can undergo a Ca^2+^ dependent switch to a pore forming conformation, and pore opening is modulated by drugs which bind to the ANT. Recent experiments on tissues from an ANT knockout mouse have thrown a question mark over this model leaving the molecular identity of the pore uncertain. It is clear, however, that pore opening is regulated by the matrix protein cyclophilin D (CypD), which binds to cyclosporine A (CsA), preventing pore opening. Protection by CsA has now become the benchmark for pore opening and is now being used in clinical trials for mPTP involvement in various pathologies. The role of the mPTP in cell death during ischaemia and reperfusion in the heart is clear and unambiguous, and infarct size is clearly reduced in the CypD knockout.^60,61^ Protection against a variety of pathologies has now been shown in the CypD knockout, including a reduction in stroke damage, and protection from experimental allergic encephalopathy.^62^ Thus identification of cell death as necrotic does not necessarily mean that the injury is untreatable.

Programmed cell death or apoptosis occurs via two signalling pathways: (i) the extrinsic pathway; which involves cell surface receptors culminating in caspase 8 activation; and (ii) the intrinsic pathway; that requires mitochondrial outer membrane permeabilization.^63^ The complex role of mitochondria in mammalian cell death was highlighted when several studies elucidated resident mitochondrial proteins were able to stimulate cell death directly.^2,63,64^ Under normal cellular conditions these proteins reside in the intermembrane space, and in response to death stimuli are released into the cytosol. They promote cell death by activating caspases and/or inactivating cytosolic inhibitors of this process. The intrinsic pathway is therefore a delicate balance between mitochondria and various cytosolic factors and it is this equilibrium that governs cellular integrity.

### Apoptogenic proteins and mitochondria

**Fig. 3:**
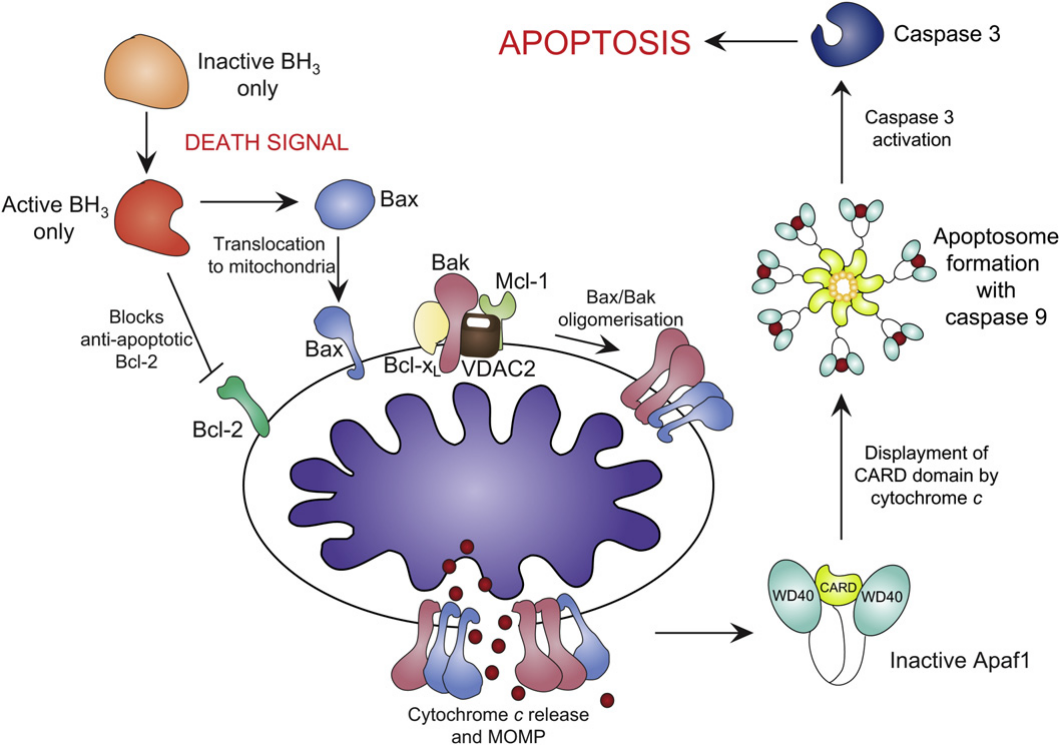
Apoptotic activation via the intrinstic pathway. Apoptotic stimuli activates the BH3-only proteins, concurrently inactivating Bcl-2 and activating Bax translocation to mitochondria. Bak is held in check by Mcl-1, VDAC2 and Bcl-xL. Bax/Bakoligomerisation results in cytochrome *c* release and MOMP. apaf-1 is activated by cytochrome *c* binding, displacing the CARD domain. The apoptosome forms with caspase-9, activating caspase-3 and triggering apoptosis.

Cytochrome *c*, an essential component of the electron transport chain initiates apoptosis when released from mitochondria.^65^ Once released, cytochrome *c* binds to Apaf-1. Further stabilisation and binding of ATP to the Apaf-1/cytochrome *c* complex results in the oligomerisation and formation of the apoptosome (Fig. 3). This multimeric complex exposes the CARD domains of Apaf-1, resulting in an open conformation. This complex is able to recruit procaspase-9, and form the active apoptosome.^66^ It is only caspase-9 that can cleave and activate the downstream executioner caspase-3. Loss of function studies in mice show that knockout of cytochrome *c* is embryonic lethal, however, at the level of a whole organism, it is difficult to distinguish whether this is largely due to its role in oxidative phosphorylation or cell death.^63^ Studies of embryonic stem cells and fibroblasts from these mice show the importance of cytochrome *c* in terms of death stimuli. In response to UV, γ-irradiation and treatment with chemotherapeutic drugs, cells failed to show caspase activity and are essentially resistant to apoptosis.^64^

Bcl-2 was the first example of an oncogene that inhibits cell death rather than promotes proliferation.^67^ The Bcl-2 family of proteins is classified into two groups, pro-survival (Bcl-x_L_, Bcl-w, A1 and Mcl-1) and pro-apoptotic (Bax, Bak, Bok, Bid, Bim, Bad, Noxa and Puma).^68,69^ The apoptogenic proteins can further be classified by the amount of Bcl-2 homology domains they contain. The BH3-only class of proteins contain a BH3 domain and amphipathic helix responsible for the interaction with the Bcl-2 family members.^70^ The majority of the BH3-only proteins translocate to the mitochondrial outer membrane upon death stimuli. The relocation to mitochondria is a critical and essential stage in cell death as it is the interaction of the BH3-only proteins with the pro-apoptotic Bcl-2 family members (Bax and Bak) that promote cell death.^70^ This translocation of the BH3-only proteins occurs simultaneously with the conformational changes and subsequent oligomerisation of Bax and Bak at the mitochondrial surface.^71^

In viable mammalian cells Bax is located in the cytosol with small amounts loosely associated with themitochondrial surface.^72^ Bax cycles on and off the outer membrane where it is retrotranslocated to the cytosol by Bcl-x_L_.^73^ This may be a regulatory checkpoint to ensure Bax levels on mitochondria do not accumulate to levels that result in auto-activation. Conversely, upon apoptotic stimuli Bax undergoes a two-step conformational change where the hydrophobic C-terminal region once concealed within the hydrophobic pocket is exposed causing the protein to translocate to mitochondria.^74^ A second conformational change occurs when the α5 and α6 helices insert directly into the outer membrane, culminating in mitochondrial outer membrane permeabilization (MOMP) and cytochrome *c* release.^75^ The mechanism that triggers Bax association with the mitochondrial surface in healthy cells is unclear, however, experiments performed with liposomes suggest that contact with the lipid bilayer may be sufficient.^76^ In addition to the Bcl-x_L_ checkpoint that prevents lethal levels of Bax accumulation on mitochondria, the composition of the outer membrane itself, namely cholesterol content may hamper the complete conformational change required to activate apoptosis.^77^ The regulation of Bax is a complex process that requires many additional proteins including the pro-apoptotic Bak. Bak is a resident protein of the mitochondrial outer membrane and is held in an inactive state by VDAC2, Mcl-1 and Bcl-x_L_.^78,79^ As with Bax, it requires BH3-only proteins to oligomerise and cause MOMP.^80^ Early in the activation process, the BH3 domain of Bak is exposed and subsequently interacts with the hydrophobic groove of another Bak molecule.^81^ It is proposed that the newly oligomerised Bax and Bak form a transitional pore, allowing apoptogenic proteins, such as cytochrome *c* to pass through, form the active apoptosome and trigger downstream executioner caspases to complete the apoptotic process.^82,83^

## Summary

Mitochondria lie at the crux of cellular viability and alterations to function are often to the detriment of the cell/organism. Mitochondria have evolved to control a diverse number of processes including cellular energy production, calcium signalling and apoptosis. Under aerobic conditions mitochondria produce energy in the form of ATP and maintain an electrochemical membrane gradient. Reduction in proton pumping across the membrane can result in decreased cell viability and induction of the intrinsic apoptotic pathway. Cytochrome *c* released from mitochondria is the point of no return in terms of cell survival as this triggers activation of apoptosis. Mitochondria are dynamic organelles and their shape is controlled by a balance of fission and fusion events. These mechanisms are tightly regulated by numerous proteins that are associated with disease states when mutations arise, highlighting the importance of organelle morphology in defining function.

Understanding the multifactorial role of mitochondrial function in cell viability, disease and inheritance is very much an evolving topic and though we are now beginning to understand the greater picture, many questions remain unanswered.

**Research agenda**

• What is the exact number of protons required to produce an ATP molecule and how many ATP molecules are produced by oxidative phosphorylation?
• What ultimately regulates both inner and outer membrane remodelling and how do these impact function?
• Which proteins/complexes comprise the apoptogenic pore in the mitochondrial membranes that facilitate cytochrome *c* release?
• What are the components of the mPTP and how are they regulated?

## Conflict of interest statement

The authors state there are no conflicts of interest.
